# Early Orthostatic Exercise by Head-Up Tilt With Stepping vs. Standard Care After Severe Traumatic Brain Injury Is Feasible

**DOI:** 10.3389/fneur.2021.626014

**Published:** 2021-04-14

**Authors:** Christian Gunge Riberholt, Markus Harboe Olsen, Christian Baastrup Søndergaard, Christian Gluud, Christian Ovesen, Janus Christian Jakobsen, Jesper Mehlsen, Kirsten Møller

**Affiliations:** ^1^Traumatic Brain Injury Unit, Department of Neurorehabilitation, Rigshospitalet, University of Copenhagen, Copenhagen, Denmark; ^2^Department of Clinical Medicine, Faculty of Health Sciences, University of Copenhagen, Copenhagen, Denmark; ^3^Department of Neuroanaesthesiology, Rigshospitalet, University of Copenhagen, Copenhagen, Denmark; ^4^Department of Neurosurgery, Rigshospitalet, Copenhagen University Hospital, Copenhagen, Denmark; ^5^Copenhagen Trial Unit, Department 7812, Centre for Clinical Intervention Research, Copenhagen University Hospital-Rigshospitalet, Copenhagen, Denmark; ^6^Department of Regional Health Research, The Faculty of Heath Sciences, University of Southern Denmark, Odense, Denmark; ^7^Department of Neurology, Bispebjerg and Frederiksberg Hospital, Copenhagen, Denmark; ^8^Surgical Pathophysiology Unit, Juliane Marie Centre, Rigshospitalet, Copenhagen University Hospital, Copenhagen, Denmark

**Keywords:** early mobilization, traumatic brain injury, feasibility trial, head-up tilt, adverse events

## Abstract

**Background:** Intensive rehabilitation of patients after severe traumatic brain injury aims to improve functional outcome. The effect of initiating rehabilitation in the early phase, in the form of head-up mobilization, is unclear.

**Objective:** To assess whether early mobilization is feasible and safe in patients with traumatic brain injury admitted to a neurointensive care unit.

**Methods:** This was a randomized parallel-group clinical trial, including patients with severe traumatic brain injury (Glasgow coma scale <11 and admission to the neurointensive care unit). The intervention consisted of daily mobilization on a tilt-table for 4 weeks. The control group received standard care. Outcomes were the number of included participants relative to all patients with traumatic brain injury who were approached for inclusion, the number of conducted mobilization sessions relative to all planned sessions, as well as adverse events and reactions. Information on clinical outcome was collected for exploratory purposes.

**Results:** Thirty-eight participants were included (19 in each group), corresponding to 76% of all approached patients [95% confidence interval (CI) 63–86%]. In the intervention group, 74% [95% CI 52–89%] of planned sessions were carried out. There was no difference in the number of adverse events, serious adverse events, or adverse reactions between the groups.

**Conclusions:** Early head-up mobilization is feasible in patients with severe traumatic brain injury. Larger randomized clinical trials are needed to explore potential benefits and harms of such an intervention.

**Clinical Trial Registration:** [ClinicalTrials.gov], identifier [NCT02924649]. Registered on 3rd October 2016.

## Introduction

Patients with severe traumatic brain injury need extensive rehabilitation ranging from their in-hospital stay at neurocritical care units over in-hospital specialized rehabilitation units to services offered after hospital discharge (1). When the condition that necessitated neurocritical care stabilizes, therapeutic efforts focus on weaning from deep sedation and mechanical ventilation rather than on mobilization. Mobilization using a tilt table in patients with impaired consciousness is typically first offered as an intervention modality after transfer out of the critical care unit to in-hospital rehabilitation wards, where the aim is to stimulate arousal and prevent secondary complications such as contractures of weight-bearing joints (2–5). In other patient categories in the critical care unit, however, early mobilization seems to be associated with positive effects on delirium, days on a ventilator, amount of sedation needed, and on functional outcome in a variety of patients in the critical care unit (6).

Orthostatic hypotension is often observed in patients with acute brain injury and could complicate the use of a tilt table for mobilization (7, 8). The pathophysiology of orthostatic hypotension is considered to be multifactorial and comprise, e.g., impaired baroreflex sensitivity, cardiovascular deconditioning, and lack of fluid retention due to neuroendocrine impairment (7, 9). Immobilization during hospitalization and the brain injury itself will facilitate the development of orthostatic hypotension (6). This will potentially hamper the recovery, either by depriving the brain of a sufficient oxygen supply during orthostatic episodes or by reducing the amount of rehabilitation offered to the patient (7). Patients with traumatic brain injury often exhibit impaired cerebral autoregulation due to increased intracranial pressure or hypotension (10). Conversely, the early orthostatic challenge may potentially prevent deconditioning by activating protective cardiovascular and neuroendocrine responses (11–13).

Early mobilization using a tilt table has been investigated in one randomized clinical trial randomizing 40 participants with different types of brain injury. They found indications of a beneficial effect on consciousness and disability at discharge from the intensive care unit and rehabilitation unit (14).

In the present trial, we investigated the feasibility of using early orthostatic exercise by a head-up tilt with stepping to mobilize to the upright position, compared to standard care in patients, who had been admitted to the neurointensive care unit with severe traumatic brain injury. We randomized participants after their brain injury who had subsided to a point where head-up tilt was deemed by the clinicians to be safe. Feasibility was assessed by whether patients could be recruited for the study and undergo the planned exercise sessions, as well as by adverse events and reactions.

## Materials and Methods

The trial protocol has previously been published (15); the statistical analysis plan was planned before the study ended (Riberholt CG et al. “Statistical analysis plan for early mobilization by head-up tilt with stepping vs. standard care after severe traumatic brain injury – a randomized clinical feasibility trial,” submitted. doi: 10.21203/rs.2.468/v3). This study was approved by the Regional Ethics Committee of the Capital Region in Denmark (H-16041794) and registered at clinicaltrials.gov (ClinicalTrials.gov identifier: NCT02924649). Patients admitted to the neurocritical care unit at University Hospital of Copenhagen - Rigshospitalet, Denmark, between January 2017 and December 2018 were screened daily by the primary investigator (CGR) and a physiotherapist. After a patient was deemed eligible for participation, informed written consent from the next-of-kin and a trial guardian (a physician not involved in the study) was obtained by a member of the trial staff.

### Participants

Patients admitted to the Neurocritical Care Unit at Rigshospitalet, Copenhagen, Denmark, with severe traumatic brain injury were eligible for inclusion and were screened daily by the primary investigator (CGR) and another physical therapist. Severe traumatic brain injury is commonly defined as a Glasgow Coma Score (GCS) <9. In this trial, we defined severe traumatic brain injury as a GCS <11 to ensure inclusion of all patients who were later diagnosed with a vegetative (unresponsive wakefulness) or minimally conscious state. In addition, inclusion criteria included a stable intracranial pressure <20 mmHg for 24 h at the time of inclusion. If the participants afterwards presented with intracranial pressure above the before mentioned limit, they were not excluded, but the exercise was canceled on that day and counted as a missing exercise. These participants received the exercise on the following day provided that intracranial pressure was below the limit. Patients were excluded if they had spinal cord injury or fractures of the lower extremities that prohibited weight-bearing, or if no informed consent was obtained. To prepare the clinicians for the tilt-table exercise the hemodynamic stability was tested in all participants to the standing position (70 degrees) before randomization.

Rigshospitalet is a 1,200-bed, tertiary-level university hospital with a level 1 trauma center. The clinical treatment of patients adhered to the most recent guidelines of the Brain Trauma Foundation (16, 17). During the study period, intracranial pressure monitoring was used as a standard throughout; monitoring of brain tissue oxygen tension underwent implementation, for patients with a GCS <9, and was not standard treatment in all patients. After the need for highly specialized neurointensive care ceases, patients are transferred either to a stepdown ward at the Department of Neurosurgery or the Department of Neurology at Rigshospitalet, or to the Department of Neurology or the Intensive Care Unit at another hospital in eastern Denmark. Referral to the Department for Highly Specialized Neurorehabilitation at Rigshospitalet, which is geographically separated from the Department of Neurosurgery and the Neurointensive Care Unit, is done at the discretion of the attending neurosurgeon. The referral is based on a clinical appraisal of the severity of the head injury and the prognosis for functional improvement in the individual patient.

### Randomization and Masking

We randomized participants when clinicians deemed head-up tilt safe, e.g., without risk of provoking intracranial pressure surges. After measurements at baseline, participants were randomly assigned (1:1) to the intervention group or the control group using a central web-based computer-generated block randomization procedure. Block sizes were randomly assigned with either 4, 6, or 8 participants in each block unknown to the investigators. We stratified the randomization according to the GCS at the time of inclusion (low GCS, 3–6 points; high GCS, 7–10 points). The randomization procedure was set up by an independent statistician at the Copenhagen Trial Unit. None of the investigators involved in the recruitment, data collection, or data analysis had access to the allocation sequence or block sizes.

Due to the nature of the intervention (tilt table), it was not possible to mask the investigators, the staff at the Neurointensive Care Unit, or the patient. Functional outcomes (as exploratory outcomes) were assessed by trained staff at the Department for Highly Specialized Neurorehabilitation. The outcome assessors assessing the *Coma Recovery Scale-Revised* and adverse and serious adverse events were blinded to the allocation of the participant, but the *Early Functional Ability Scale* and the *Functional Independence Measure* were assessed by the department staff without masking.

### Interventions

The intervention group underwent early orthostatic exercise and otherwise received the same treatment throughout as the control group. The early orthostatic exercise consisted of daily (Monday to Friday) exercise on an ERIGO basic® tilt-table (Hocoma AG, Switzerland) to 70° head-up tilt for 20 min administered by two physiotherapists and a nurse. The ERIGO basic® has a built-in robotic stepping device intended to counteract a drop in blood pressure during standing (8). The robotic stepping frequency was set at 50–60 steps per minute. If reduction in blood pressure, cerebral perfusion pressure or increase in heart rate and intracranial pressure beyond predetermined limits (15) were observed, the participants were moved to the supine position until stable and then returned to head-up tilt. Time spent in the supine position did not count in the duration of the daily exercise session. The orthostatic exercise sessions were terminated if participants regained the ability to stand up by themselves, but they remained in their assigned group.

The control group received standard treatment at the department, as decided by the treating physicians, nurses, and therapists. This treatment followed recommendations from the Brain Trauma Foundation (16). Standard treatment included mobilization but to a much smaller scale, and the focus of the physical therapists was primarily on respiratory function and re-positioning to avoid bedsores. Mobilization was done to the edge of the bed or by lifting the patient to a (wheel or comfort) chair. The mobilization in the standard-care group did not include regular mobilization on a tilt table. However, both groups underwent a hemodynamic test during head-up tilt without stepping before randomization, and at 2- and 4-week follow-up.

### Outcomes

The primary outcome focused on the feasibility of the study and consisted of a combination of the following three measures of feasibility and safety: 1. The number of included participants relative to the total number of the eligible patients. For the study to be feasible, we required that the lower 95% confidence limit of this number was at least 60%. 2. The number of exercise sessions we were able to perform relative to the planned number. As described in our protocol, we required that 60% of the intended exercises be completed. For the study to be feasible, the lower 95% confidence limit of the proportion of participants that completed 60% of the intended exercises was required to be at least 52%. These limits correspond to a one-sided significance test of 0.025. 3. The total number of serious adverse events and reactions as well as adverse events and reactions in each group at the end of the 4-week intervention period. For acceptable safety, the number in the intervention group was required not to exceed that in the control group. Thus, the study was successful if all three requirements were fulfilled, as also stated in the statistical analysis plan.

An adverse event (AE) is defined as any undesirable event not considered serious occurring to a participant during the trial. A severe adverse event (SAE) is defined as any undesirable event that results in death, is life-threatening, requires prolongation of existing hospitalization, results in persistent or significant disability or incapacity, or requires intervention to prevent permanent impairment or damage, whether considered related to the trial intervention or not. An adverse reaction (AR) is defined as above but it is directly related to the intervention that is investigated—in this case, mobilization in a tilt table. Severe adverse reactions (SAR) is defined as SAE but is directly related to the intervention that is being investigated– in this case, mobilization in a tilt-table (18).

As exploratory clinical outcomes, we registered any suspected unexpected serious adverse reactions and measured the Coma Recovery Scale-Revised, Early Functional Ability Scale and Functional Independence Measure. All exploratory clinical outcomes were assessed at baseline, and after 4 weeks, 3 months, and 1 year.

If participants were transferred to other departments within the hospital, they were followed up until 1 year after the original injury.

### Statistical Analysis

We estimated the trial power pragmatically to include 60 participants (15). However, we did not reach this number, resulting in an inadvertently lower power for our trial.

Continuous baseline characteristics are presented as either means and standard deviations (SD) for normally distributed data or medians and interquartile ranges (IQR) for non-normally distributed data. Ordinal variables are presented as medians and interquartile ranges. Discrete variables are presented as frequencies, proportions, and percentages.

The ratio of the two feasibility outcomes was calculated as Wilsons interval, and Jeffreys interval with a 95% confidence interval (CI) as these are recommended for proportions from small populations (19). The Jeffrey interval is based on a Bayesian distribution of 0.5 and the Wilson interval on a normal distribution (19). If there is a large difference between the two, the most conservative lower confidence interval was used to determine if the trial procedure was feasible. Adverse events, serious adverse events and adverse reactions were analyzed between groups using Fisher's exact test. We did not use the originally planned logistic regressions analysis due to splitting in data and a high proportion of participants with one or more events. A descriptive analysis of the most common serious adverse events and adverse events not considered serious are presented as frequencies and percentages by each group.

For the exploratory outcomes, the analysis was primarily intention-to-treat using the van Elteren's test for non-normally distributed data, stratified for GCS. As a sensitivity analysis, we did a per-protocol analysis using the participants in the early orthostatic exercise group that completed at least 60% of the intended interventions. Trial Sequential Analysis was used to quantify the reliability of the statistical analysis and determine the required information sizes (Trial Sequential Analysis. Copenhagen Trial Unit, 2011) (20–22). All statistical analyses were carried out in Stata 15 (StataCorp, TX, USA).

## Results

**Figure 1:**
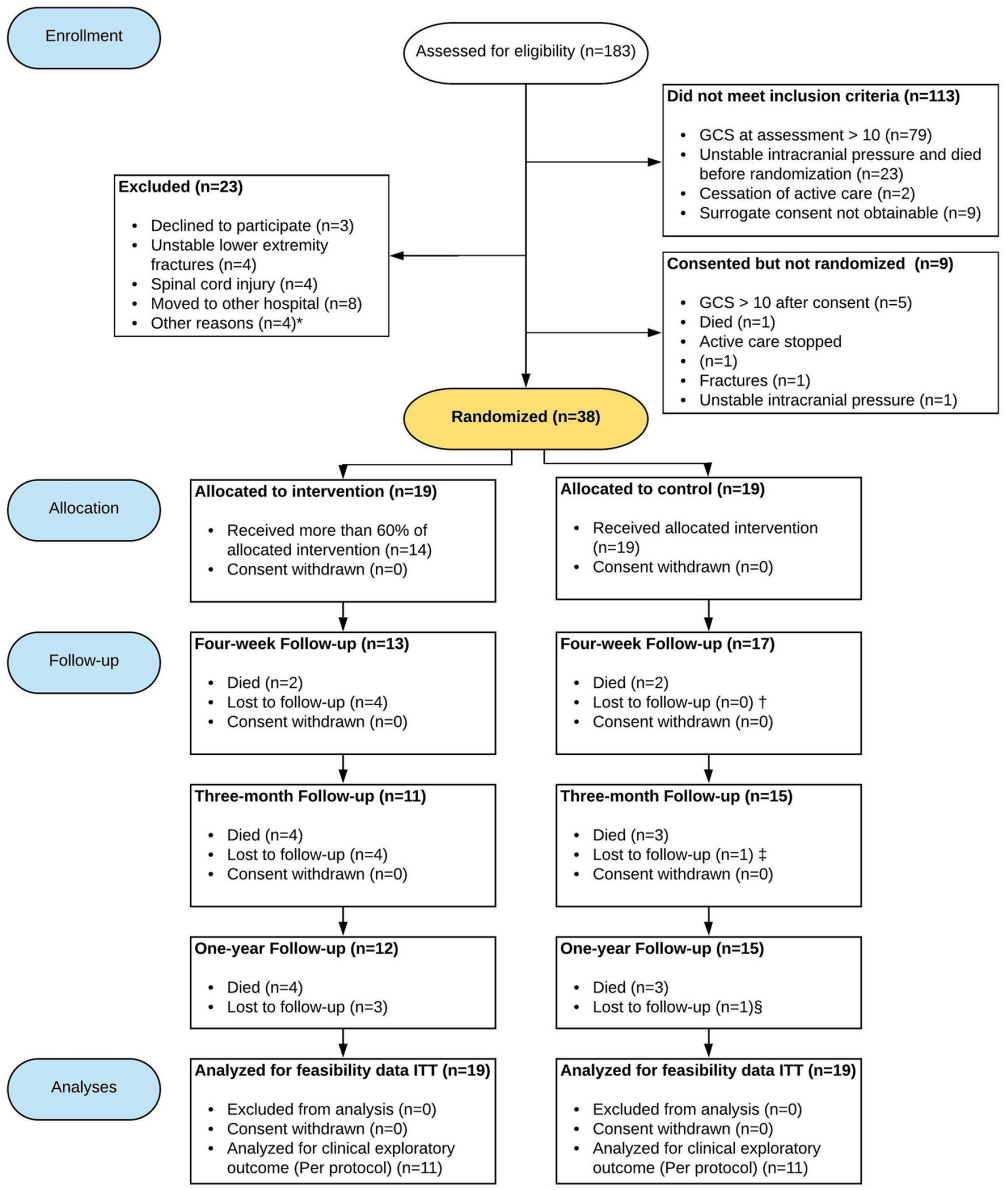
Flow of patients through the trial. GCS, Glasgow coma score; ITT, Intention to treat. *Other reasons include: High frequency of dialysis, waiting for a pacemaker, body weight exceeding the limit of the mobilization device; ^†^Coma Recovery Scale-Revised (*n* = 16) one patient discharged before the test; ^‡^Early discharge from the rehabilitation department before assessments (*n* = 1); ^§^In the intervention group one patient was not assessed with Coma Recovery Scale-Revised and Early Functional Ability and in the standard care group one patient was not assessed with Coma Recovery Scale-Revised. Due to the nature of the Glasgow Outcome Scale Extended (where 1 equals death), all participants were scored in the standard care group. In the intervention group, three were lost to follow-up.

During the intervention period, 50 patients were eligible for inclusion. Three declined to participate; for 47 patients, the next of kin provided informed consent. This gave a consent proportion of 94% [95% CI: 84–98%]. Nine of the 47 patients were not able to be included due to improvement in neurological status (*n* = 5), death (*n* = 1), cessation of active care (*n* = 1), continuous unstable intracranial pressure (*n* = 1) between the time of consent and randomization, or fractures discovered after consent was given (*n* = 1) (Figure 1). Therefore, 38 participants were included with a mean (SD) delay of 13 (5) days after injury (19 participants in the intervention group and 19 in the standard care group) (Table 1). Thus, 76% [95% CI: 63–86%] of all eligible patients eventually participated in the trial (Table 2).

**Table 1:** Baseline characteristics of included participants.

	**Early orthostatic exercise (*n* = 19)**	**Standard care group (*n* = 19)**
Age (years) – median (IQR)	49.0 (31.0–63.0)	37.0 (27.0–54.0)
Male – *n* (%)	13 (68%)	14 (74%)
Brain injury (initial CT-scan) – *n* (%)		
Traumatic subarachnoid hematoma	10 (53%)	17 (89%)
Acute subdural hematoma	14 (74%)	17 (89%)
Chronic subdural hematoma	1 (5%)	2 (11%)
Epidural hematoma	3 (16%)	3 (16%)
Intraventricular hematoma	10 (53%)	3 (16%)
Contusion	12 (63%)	11 (58%)
Mechanism of injury – *n* (%)		
Traffic	8 (42%)	9 (47%)
Fall	8 (42%)	6 (32%)
Blunt force	1 (5%)	4 (21%)
Penetrating	1 (5%)	-
Unknown	1 (5%)	-
Secondary injury – *n* (%)		
1 fracture of extremities or trunk	4 (21%)	4 (21%)
>1 fracture of extremities or trunk	6 (32%)	4 (21%)
No fractures	9 (47%)	11 (58%)
Comorbidities – *n* (%)		
Diabetes (type II)	1 (5%)	-
Pulmonary heart disease	1 (5%)	-
Hypertension	-	1 (5%)
Schizophrenia	2 (11%)	-
Chronic obstructive lung disease	1 (5%)	1 (5%)
Atrial fibrillation	1 (5%)	1 (5%)
None	16 (84%)	17 (95%)
Neurosurgical procedures performed – *n* (%)		
Evacuation of hematoma	8 (42%)	7 (37%)
Craniotomy	9 (47%)	9 (47%)
Craniectomy	4 (21%)	6 (32%)
External ventricular drain	13 (68%)	14 (74%)
Ventriculoperitoneal shunt	3 (16%)	1 (5%)
First measured GCS – median (IQR)	6 (3 to 9)	6 (3 to 9)
Low GCS (3 to 6) – *n* (%)	10 (53%)	10 (53%)
GCS at inclusion - median (IQR)	4 (3 to 4)	5 (3 to 6)
High GCS (7 to 10) – *n* (%)	9 (47%)	9 (47%)
GCS at inclusion - median (IQR)	7 (7 to 7)	9 (9 to 9)
Sedated at randomization – *n* (%)	6 (32%)	6 (32%)
RASS– median (IQR)	−3 (−4 to −3)	−5 (−5 to −3)
Days from injury to randomization – median (IQR)	15 (11 to 16)	10 (7 to 14)
Days to first mobilization - median (IQR)	15 (11 to 16)	12 (10 to 18)^*^
Days at the Neuro Critical Care Unit – median (IQR)	32 (22 to 40)	25 (18 to 34)
Days at the RU – median (IQR)	72 (37–99)	67 (46–79)
End of PTA (days) – median (IQR)	81 (53–101)	67 (39–99)

* *One patient in the standard care group never received mobilization*.

*SD, Standard deviation; n, number; GCS, Glasgow coma score; IQR, Interquartile range; RASS, Richmond agitation sedation scale; RU, Rehabilitation unit; PTA, Posttraumatic amnesia*.

**Table 2:** Feasibility outcome.

	**n/N (% [95% CI]) *(Wilson confidence interval)***	**n/N (% [95% CI]) *(Jeffreys confidence interval)***
Included participants	38/50 (76.0% [62.6–85.7%])	38/50 (76.0% [62.9–86.2%])
Participants with > 60% completed exercises	14/19 (73.7% [51.2–88.2%])	14/19 (73.7% [51.6–89.2%])
	**Early orthostatic exercise (*****n*** **= 19 participants)**	**Standard care group (*****n*** **= 19 participants)**
Orthostatic exercise sessions – mean (±SD)	10.7 (5.9)	-
Additional mobilizations – median (IQR)	3 (0–9)	8 (3 to 16)^*^
Additional mobilizations by nurses – median (IQR)	0 (0;0)^†^	0 (0;1)^‡^

* *Two standard-care patients had more than 70 mobilizations during the intervention period*.

† *One intervention patient was mobilized 9 times*.

‡ *Five standard-care patients were mobilized between 1 and 15 times*.

*N, All approached patients; 95%CI, 95% confidence interval; SD, standard deviation; IQR, interquartile range*.

Of the 19 participants in the early orthostatic intervention group, 14 (74% [95% CI: 51.6–89.2%], Jeffreys interval) received at least 60% of the intended exercise sessions (Table 2).

Of the 38 included participants, four were transferred out of the participating hospital to critical care units, rehabilitation units or psychiatric wards within the 4-week intervention period. These hospitals not participating in the trial and consequently the participants were lost to follow-up. Furthermore, two participants died, and another two participants had their active care stopped due to an expected poor prognosis. None of the participants withdrew their consent to participate during the trial period.

In the intervention group, a total of 203 exercise sessions were completed corresponding to an average of 11 sessions per participant (Table 2). Of the sessions that were not completed the main reasons were transfer to other departments (60%), cessation of active care (5%), fever (4%), removal of tracheal tube (3%), and agitation (3%). Less common reasons were percutaneous endoscopic gastronomy, high intracranial pressure, need of acute radiology, unstable hemodynamics, vomiting, other operational procedures prioritized, and malfunction of the tilt-table. A total of 46 orthostatic reactions corresponding to two (median; IQR, 0 to 3) orthostatic reactions per patient in the intervention group occurred. Eight of the 19 participants experienced no orthostatic reactions.

During the 4-week intervention period, we registered a total of 202 adverse events or reactions. Forty-six were serious adverse events, and seven were adverse reactions that occurred either during the tilt-table intervention or (for the standard-care group) the hemodynamic test. No serious adverse reactions occurred. Table 3 shows the distribution of adverse events and reactions in the two intervention groups. For a complete list of serious and non-serious adverse events, please refer to Supplementary Table 3. We found no statistically significant difference between participants in the two groups experiencing at least one adverse event, serious adverse event, or adverse reaction (Tables 3, 4).

**Table 3:** Adverse events and reactions during the 4-week intervention period.

	**Early orthostatic exercise (*n* = 19 participants)**	**Standard care group (*n* = 19 participants)**	***P*-value**
**Patients experiencing at least one** Adverse events – *n* (%)	17 (89)	17 (89)	1.000
Serious adverse events – *n* (%)	14 (74)	13 (68)	1.000
Adverse reactions – *n* (%)	1 (5)	3 (16)	0.604
Serious adverse reactions – *n* (%)	-	-	
SUSAR – *n* (%)	-	-	
**Total number of events** Adverse events (*n* = 149) – *n* (%)	73 (49)	76 (51)	
Serious adverse events (*n* = 46) – *n* (%)	24 (52)	22 (48)	
Adverse reactions (*n* = 7) – *n* (%)	4 (57)	3 (43)	
Serious adverse reactions (*n* = 0) – *n* (%)	-	-	
SUSAR (*n* = 0) – *n* (%)	-	-	

*n, number; SUSAR, Suspected unexpected serious adverse reaction*.

**Table 4:** Adverse events and reactions during the 4-week intervention period – Per protocol analysis.

	**Early orthostatic exercise (*n* = 14 participants)**	**Usual care group (*n* = 19 participants)**	***P*-value**
**Patients experiencing at least one** Adverse events – *n* (%)	13 (93)	17 (89)	1.000
Serious adverse events – *n* (%)	11 (79)	13 (68)	0.698
Adverse reactions – *n* (%)	1 (7)	3 (16)	0.620
Serious adverse reactions – *n* (%)	-	-	
SUSAR – *n* (%)	-	-	
**Number of events** Adverse events (*n* = 134) – *n* (%)	58 (43)	76 (57)	
Serious adverse events (*n* = 41) – *n* (%)	19 (46)	22 (54)	
Adverse reactions (*n* = 7) – *n* (%)	4 (57)	3 (43)	
Serious adverse reactions (*n* = 0) – *n* (%)	-	-	
SUSAR (*n* = 0) – *n* (%)	-	-	

*n, number; SUSAR, Suspected unexpected serious adverse reaction*.

The Trial Sequential Analysis of serious adverse events and adverse events showed that a required information size of 628 and 243 participants would be needed to reach the required information size, respectively (Supplementary Figures 1A,B); i.e., a total of 628 participants would need to be included in a trial to draw a reliable conclusion on the risk of serious adverse events in the intervention compared to the standard-care group, whereas a total of 243 participants would be necessary for the same analysis regarding adverse events. For the current trial, the risk of serious adverse events and adverse events in the intervention group did not differ from that of the control group; the diversity-adjusted Trial Sequential Analysis confidence interval for the relative risk of the intervention group ranged from 0.2 to 5.7 for serious adverse events and from 0.5 to 2.0 for adverse events.

### Exploratory Outcomes

No suspected unexpected serious adverse reactions were registered during the trial.

**Figure 2:**
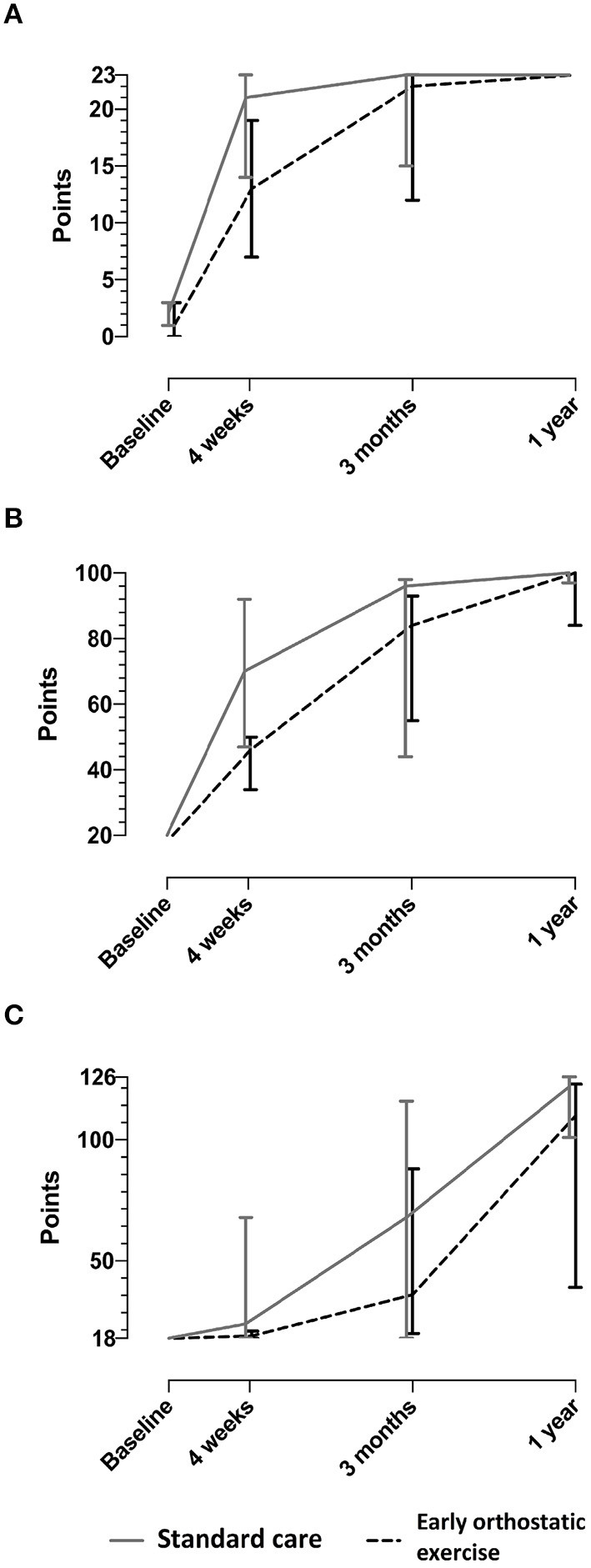
Exploratory outcomes. The figure displays the median score with interquartile ranges. Raw data are presented in Supplementary Table 1. **(A)** Coma recovery scale-revised (CRS-R). **(B)** Early functional ability (EFA). **(C)** Functional independence measure (FIM).

After 4 weeks, there was a trend toward less functional improvement for the intervention group (end of intervention) [Coma Recovery Scale-Revised median score, 13 (IQR: 7–9) points] compared to the control group [21 (IQR: 14–23) points] (*P* = 0.07) (Figure 2). At 3 months, the intervention group had an Early Functional Ability score of 84 [IQR: 55–93] points compared to the control group [96 (IQR: 44–98) points] (*P* = 0.24). Also, at 3 months, the intervention group achieved a Functional Independence Measure score [median 36 (IQR: 20–88) points] that did not differ compared to the control group [median 68 (IQR: 18–116) points] (*P* = 0.19). The Glasgow Outcome Scale Extended at 1-year follow-up showed no between-group differences (Supplementary Table 1). Per protocol analysis (participants with more than 60% completed exercises) showed no difference in any of the outcomes (Supplementary Table 2).

Diversity-adjusted Trial Sequential Analysis of data from the Coma Recovery Scale-Revised at 4 weeks suggested that 266 participants with severe traumatic brain injury were needed to reach the required information size. As Trial Sequential Analysis can assess a more realistic CI, a mean difference of 1.1 points was found between groups, and the Trial Sequential Analysis-adjusted CI showed a range from−16.0 to 24.4 points (Supplementary Figure 1C). In contrast, it was not possible to carry out Trial Sequential Analysis on Early Functional Ability scale or Functional Independence Measure because of too little information and a large variance in the data.

## Discussion

This trial investigated both feasibility, safety, and clinical outcomes from early orthostatic exercise in participants with severe traumatic brain injury. Inclusion into the trial was accepted by relatives of the participants at a high percentage (94%), and 76% were randomized. Following randomization, we managed to deliver 74% of the intended interventions with a lower confidence limit at 51.6%. We found no differences between groups with respect to adverse events or reactions and on our exploratory outcomes (Coma Recovery Scale—Revised, Early Functional Ability Scale and Functional Independence Measure).

The confidence limits of the feasibility outcomes may inform future trials on what to expect regarding inclusion rate and successful delivery of exercises. A lower boundary in future trials of successfully delivered interventions down to 50% may not be an acceptable rate. In the original protocol for the ethics committee a limit was set at 80% completed exercises. During the drafting of the protocol article (15), we decided to change this limit to 60% to mimic the recommendations on physical activity from the Danish health authorities. The change was therefore made before the majority of patients were included. In the present study, the main reason for not completing exercises was patient transfer to departments that were not included in the study. This challenge obviously depends on how healthcare is organized in the catchment area; similar studies may benefit from careful preceding analysis of patient flow and contingency planning to ensure a high patient retention rate, which not only is critical to the resulting power of the study but also helps avoid attrition bias.

The present trial suggests that early head-up mobilization may not increase the risk of harm. This may be at odds with the largest trial so far on mobilization of patients with acute stroke (*N* = 2,104), which found that early mobilization decreased the odds ratio of reaching a favorable outcome (OR 0.73; 95% CI 0.59–0.90) (23), although a prespecified dose-response analysis showed an improved outcome after 3 months if participants initiated early rehabilitation with higher frequency but shorter duration of sessions (24). Nonetheless, these patients are not immediately comparable with the patients included in our present trial, as the latter were, in general, deeply sedated for many days before undergoing mobilization; in the former trial, patients were generally not sedated and started mobilization within the first 24 h. We did not investigate whether adverse events, serious adverse events, adverse reactions or serious adverse reactions prohibited the exercises. This could be relevant in a larger trial investigating this type of exercise.

Studies and trials investigating early mobilization in participants with acute brain injury have generally reported diverting results. A previous pilot study on patients with acute brain injury (stroke, traumatic brain injury, etc.) mobilized participants starting a mean of 12 (SD 7) days after injury using the same technique (14). The authors included 20 participants in both the intervention and the control group and reported no adverse events; five participants in the intervention group and four in the control group died (14). The study found a significant beneficial effect of early mobilization on the Coma Recovery Scale-Revised and the Disability Rating Scale after 1 month and ~4 months (14). A quasi-randomized study of 61 patients with traumatic brain injury also reported a clinical benefit of starting mobilization in the intensive care unit, although selection bias cannot be ruled out (25). Finally, two trials focusing on early mobilization conducted in the intensive care unit showed improved functional outcome at hospital discharge (6, 26), but only a few of these participants had a traumatic brain injury. Although at first sight the findings of these smaller studies differ from that of our present trial, these should be considered underpowered to draw any conclusion on benefits or harms. Thus, the Trial Sequential Analysis in the present trial of patients with traumatic brain injury indicated that a total sample size of more than 600 participants would be needed for firm conclusions on harms or benefits from early mobilization. We have recently published a systematic review on early mobilization of patients with severe acquired brain injury showing insufficient evidence of the benefits and harms for this treatment (27).

The present trial has several limitations. We did not reach the desired number of participants as recruitment was stopped at the end of the planned inclusion period (2 years). The recruitment rate was lower than expected, which could be partly explained using rather narrow limits for the Glasgow Coma Scale at the time of inclusion. At any rate, the sample size estimate was pragmatic. Our trial was small, and such small trials run the risk of uneven distribution of prognostic factors. We did not statistically test the difference in the baseline data, since such tests may cause spurious results and are susceptible to multiple testing issues (28). In accordance, patients in the intervention group tended to be older than those in the control group; because lower age is associated with better outcome (29), this may have skewed the data toward more favorable outcomes in the control group. Furthermore, the high Glasgow Coma Scale strata seemed to have a lower median score in the early mobilization group compared with standard care group. Moreover, our control group was mobilized earlier than the intervention group, although this was not significantly different. Both could be an expression of a more stable condition in the control group as we used the intracranial pressure measurements as an indicator for when to initiate the intervention. There was a large amount of missing data on the clinical outcome, which was mostly due to death or transfer to other departments. We elected not to use multiple imputation in the exploratory outcomes, as we consider these results as hypothesis-generating only. Finally, as tools for functional outcome assessment, we elected to use the Coma Recovery Scale-Revised, The Early Functional Ability scale, and the Functional Independence Measure; although remote scoring could be considered for patients that were transferred out of participating hospitals, such scores were deemed insufficient as they would provide only a rough estimate of the patient's ability to function independently. While the Coma Recovery Scale-Revised seems only useful for measuring shorter-term outcomes (4 weeks), the Early Functional Abilities Scale and the Functional Independence Measure measured changes at 3 months without reaching a maximum score limit.

## Conclusion

Early orthostatic exercise is feasible in participants with severe traumatic brain injury. We did not find any certain differences between the two groups regarding benefits or harms, and larger randomized clinical trials are needed to analyze potential benefits and harms of such an intervention.

## Data Availability Statement

The raw data supporting the conclusions of this article will be made available by the authors, without undue reservation.

## Ethics Statement

The studies involving human participants were reviewed and approved by The Regional Ethics Committee of the Capital Region in Denmark (H-16041794). Written informed consent was not provided because patients were unable to. Informed consent was primarily given by next-of-kin and trial guardian. If patients regained the ability to consent, new formal consent was given by the patient.

## Author Contributions

CGR, JM, KM, and CG designed the trial. CR, MHO, and CBS collected the data. CR, CO, and JCJ analyzed the data under the supervision of CG. CR drafted the manuscript. All authors were involved in the interpretation of the results, revised it critically, and approved the manuscript.

## Conflict of Interest

The authors declare that the research was conducted in the absence of any commercial or financial relationships that could be construed as a potential conflict of interest.
