# Supplementary material for: Early Orthostatic Exercise by Head-Up Tilt With Stepping vs. Standard Care After Severe Traumatic Brain Injury Is Feasible
Source: Front Neurol. 2021 Apr 14;12:626014. doi: 10.3389/fneur.2021.626014 (PMC8079637; doi:10.3389/fneur.2021.626014)
Supplement: Supplementary file 1 [file Data_Sheet_1.docx]

# Supporting material for

Early orthostatic exercise by head-up tilt with stepping versus standard care after severe traumatic brain injury is feasible.

Christian Gunge Riberholt, Markus Harboe Olsen, Christian Baastrup Søndergaard, Christian Gluud, Christian Ovesen, Janus Christian Jakobsen, Jesper Mehlsen, Kirsten Møller

# Table of contents

- Supplementary Figure 1. Trial Sequential Analysis
- Supplementary Table 1. Exploratory clinical outcome for baseline, end of the intervention, three months and one year – Intention-to-treat
- Supplementary Table 2. Exploratory clinical outcome for baseline, end of the intervention, three months and one year – Per protocol
- Supplementary Table 3. Specification of serious adverse events and adverse events
- Supplementary material (Statistical analysis)
- CONSORT 2010 checklist of information to include when reporting a pilot or feasibility trial
- TIDieR (Template for Intervention Description and Replication) Checklist

**Supplementary figure 1. Trial Sequential Analysis.**

Legend: Post-hoc Trial Sequential Analysis of the trial results after 4 weeks (SAE, AE or AR and Coma Recovery Scale-Revised). A and B: Shows that 38 participants were assessed for having at least one SAE or at least one AE during the treatment period. The required information size of 628 participants is calculated based on the incidence in the standard care group of 68.4% (A) and 243, with an incidence of 89.5% (B). A type I error of 2.5%, a beta of 10% (power of 90%), and a relative risk reduction of 20% was used. C: shows that 29 participants were tested with the CRS-R after 4 weeks. The required information size of 266 participants is calculated based on a minimal relevant mean difference of 3 points and a standard deviation of 6 points. The analysis was based on a type I error of 2.5% and a beta of 10% (power of 90%). The cumulated Z-curves (blue curve) do not cross the trial sequential boundaries (red inner sloping lines) implying that there is a risk of random error (either due to sparse data or repetitive testing) in the estimate of a beneficial effect of early orthostatic exercise compared with standard care. Furthermore, the Trial Sequential Analysis- adjusted 95% CI (E) shows a wide range for all outcomes.

| **Supplementary Table 1. Exploratory clinical outcome for baseline, end of the intervention, three months and one year – Intention-to-treat** | Usual care group | One-year follow-up | 14 | 23 | (23 to 23) | 15 | 100 | (97 to 100) | 15 | 122 | (101 to 126) | 19 | 3 | (3 to 6) ‡ | CRS-R: Coma recovery scale - revised; EFA: Early Functional Ability; FIM: Functional independence measure; GOS-E: Glasgow outcome scale - extended; N: number of participants in the analysis; IQR: Interquartile range; For analysis Van Elteren’s test have been used adjusted for stratification variable (Glasgow Coma Score high or low at randomization):  * *P*=0.07; † *P*=0.24; ‡ *P*=0.19; |
| --- | --- | --- | --- | --- | --- | --- | --- | --- | --- | --- | --- | --- | --- | --- | --- |
|  |  | Three months from injury | 15 | 23 | (15 to 23) | 15 | 96 | (44 to 98) † | 15 | 68 | (18 to 116) ‡ |  |  |  |  |
|  |  | End of intervention | 16 | 21 | (14 to 23) * | 17 | 70 | (47 to 92) | 17 | 24 | (18 to 68) |  |  |  |  |
|  |  | Baseline | 19 | 2 | (1 to 3) | 19 | 20 | (20 to 20) | 19 | 18 | (18 to 18) |  |  |  |  |
|  | Early orthostatic exercise | One-year follow-up | 11 | 23 | (23 to 23) | 12 | 100 | (84 to 100) | 11 | 110 | (39 to 123) | 16 | 3 | (1 to 5) ‡ |  |
|  |  | Three months from injury | 11 | 22 | (12 to 23) | 11 | 84 | (55 to 93) † | 11 | 36 | (20 to 88) ‡ |  |  |  |  |
|  |  | End of intervention | 13 | 13 | (7 to 19) * | 13 | 46 | (34 to 50) | 13 | 19 | (18 to 21) |  |  |  |  |
|  |  | Baseline | 19 | 1 | (0 to 3) | 19 | 20 | (20 to 20) | 19 | 18 | (18 to 18) |  |  |  |  |
|  |  |  | **CRS-R** (N) | Median | (IQR) | **EFA** (N) | Median | (IQR) | **FIM** (N) | Median | (IQR) | **GOSE** (N) | Median | (IQR) |  |

| **Supplementary Table 2. Exploratory clinical outcome for baseline, end of the intervention, three months and one year – Per protocol** | Usual care group | One-year follow-up | 14 | 23 | (23 to 23) | 15 | 100 | (97 to 100) | 15 | 122 | (101 to 126) | 19 | 3 | (3 to 6) § | CRS-R: Coma recovery scale - revised; EFA: Early Functional Ability; FIM: Functional independence measure; GOS-E: Glasgow outcome scale - extended; N: number of participants in the analysis; IQR: Interquartile range; For analysis Van Elteren’s test have been used adjusted for stratification variable (Glasgow Coma Score high or low at randomization):  * *P*=0.06; † *P*=0.30; ‡ *P*=0.26; § *P*=0.27 |
| --- | --- | --- | --- | --- | --- | --- | --- | --- | --- | --- | --- | --- | --- | --- | --- |
|  |  | Three months from injury | 15 | 23 | (15 to 23) | 15 | 96 | (44 to 98) † | 15 | 68 | (18 to 116) ‡ |  |  |  |  |
|  |  | End of intervention | 16 | 21 | (14 to 23) * | 17 | 70 | (47 to 92) | 17 | 24 | (18 to 68) |  |  |  |  |
|  |  | Baseline | 19 | 2 | (1 to 3) | 19 | 20 | (20 to 20) | 19 | 18 | (18 to 18) |  |  |  |  |
|  | Early orthostatic exercise | One-year follow-up | 9 | 23 | (23 to 23) | 10 | 100 | (92 to 100) | 9 | 110 | (64 to 123) | 13 | 3 | (1 to 5) § |  |
|  |  | Three months from injury | 9 | 22 | (12 to 23) | 9 | 86 | (56 to 93) † | 9 | 36 | (20 to 88) ‡ |  |  |  |  |
|  |  | End of intervention | 12 | 13 | (7 to 18) * | 12 | 45 | (34 to 52) | 12 | 19 | (18 to 21) |  |  |  |  |
|  |  | Baseline | 14 | 2 | (0 to 4) | 14 | 20 | (20 to 20) | 14 | 18 | (18 to 18) |  |  |  |  |
|  |  |  | **CRS-R** (N) | Median | (IQR) | **EFA** (N) | Median | (IQR) | **FIM** (N) | Median | (IQR) | **GOSE** (N) | Median | (IQR) |  |

**Supplementary Table 3. Specification of serious adverse events and adverse events**

|  | **Early orthostatic exercise** | **Usual care group** |
| --- | --- | --- |
| **Serious adverse events** |  |  |
| Pneumonia | 9 | 7 |
| Delirium | 1 | 4 |
| Death | 2 | 2 |
| Sepsis | 4 | 1 |
| Blocked tracheal tube | 2 | 1 |
| Seizure | 2 | 0 |
| Pleural effusion | 0 | 2 |
| Ventriculitis | 1 | 0 |
| Paroxysmal sympathetic hyperactivity | 0 | 1 |
| Desaturation | 1 | 0 |
| Respiratory secretion (atelectasis) | 1 | 0 |
| Urinary tract infection | 0 | 1 |
| Agitated | 0 | 1 |
| Other infections | 0 | 1 |
| Progression of subdural hematoma | 0 | 1 |
| Sudden high intracranial pressure | 1 | 0 |
| **Total** | **24** | **22** |
|  |  |  |
| **Adverse events not considered serious** | **Early orthostatic exercise** | **Usual care group** |
| Removal of nasogastric tube | 6 | 7 |
| Pressure ulcer | 6 | 3 |
| Urinary tract infection | 3 | 5 |
| Vomiting | 3 | 5 |
| Other infections | 3 | 5 |
| Paroxysmal sympathetic hyperactivity | 3 | 3 |
| Withdrawal symptoms | 5 | 1 |
| Anemia | 2 | 3 |
| Diarrhea | 2 | 3 |
| Oral mycosis | 2 | 2 |
| Wounds | 3 | 1 |
| Hyponatremia | 1 | 3 |
| Hypokalemia | 3 | 1 |
| Fall | 1 | 2 |
| Tachycardia | 1 | 2 |
| Confusion | 3 | 0 |
| Conjunctivitis | 2 | 1 |
| Bleeding from a surgical wound | 1 | 2 |
| Blocked tracheal tube | 0 | 2 |
| Ventriculitis | 0 | 2 |
| Removal of venous or arterial catheter | 0 | 2 |
| Restless | 0 | 2 |
| Hypertension | 2 | 0 |
| hypercapnia | 1 | 1 |
| Hypernatremia | 1 | 1 |
| Hyperkalemia | 2 | 0 |
| Rash | 0 | 2 |
| Tongue biting | 1 | 1 |
| Desaturation | 0 | 1 |
| Respiratory secretion (atelectasis) | 1 | 0 |
| Hypotension | 0 | 1 |
| Agitated | 0 | 1 |
| Removal of wound dressing | 0 | 1 |
| Calf pain | 0 | 1 |
| Bleeding urethra | 1 | 0 |
| Removal of tracheotomy | 1 | 0 |
| Alkalosis | 1 | 0 |
| Hypermagnesemia | 1 | 0 |
| Hyperglycemia | 1 | 0 |
| Subcutaneous emphysema | 0 | 1 |
| Heart murmur | 0 | 1 |
| Displacement of fracture | 0 | 1 |
| Epidermolysis arm | 0 | 1 |
| Fever without origin | 0 | 1 |
| Increased saliva | 0 | 1 |
| Obstipation | 0 | 1 |
| Sleep apnea | 0 | 1 |
| Gastrointestinal bleeding | 0 | 1 |
| Acute Tubulointerstitial Nephropathy | 1 | 0 |
| Thrombocytosis | 1 | 0 |
| broken tooth | 1 | 0 |
| Loose external ventricular drain screw | 1 | 0 |
| Dysfunctional arterial catheter | 1 | 0 |
| Distended anal sphincter | 1 | 0 |
| Pancreatitis | 1 | 0 |
| Nose bleeding | 1 | 0 |
| Hematoma lower extremity | 1 | 0 |
| Joint swelling | 1 | 0 |
| **Total** | **73** | **76** |
|  |  |  |
| **Adverse reactions not considered serious** | **Early orthostatic exercise** | **Usual care group** |
| Friction wounds around ERIGO® cuffs | 3 | 0 |
| Leak of urine catheter | 1 | 1 |
| Leak of fecal bag | 0 | 1 |
| Disconnected Licox transmitter | 0 | 1 |
| **Total** | **4** | **3** |

**Supplementary material (Statistical analysis)**

***Retrospective changes to the dataset***

Post-hoc changes in the dataset after blinded assessors had gone through medical records.

- Patient 3 was diagnosed with sepsis which initially was categorized as an adverse event not considered serious. This was changed to a serious adverse event.
- Patient 5, 8 and 28 had a missing SAE as they were moved from the department and died afterwards all within the 4-week period. One SAE was added to each patient.
- Patient 24 was diagnosed with pneumonia which initially was categorized as an adverse event not considered serious. This was changed to a serious adverse event.

***Statistical analysis workflow***

First version of the statistical analysis plan was submitted to Trials on the 20^th^ March 2019, the second version on 14^th^ of June 2019 and the third on 23^rd^ of December 2019. Alas, the statistical analysis plan was rejected for publication. Since March 2019 it has been given a digital object identifying (DOI) number ([10.21203/rs.2.468/v3](https://dx.doi.org/10.21203/rs.2.468/v3)). The last 3 months follow-up was gathered on the 28^th^ of March 2019 and the last one-year follow-up on the 10^th^ of November 2019. The first data analysis of the feasibility outcomes and the exploratory clinical outcomes (including the 3-month follow-up) was done on the 7^th^ of May 2019. Final analysis of data including the one-year follow-up was done on the 28^th^ of January 2020. On the 28^th^ of February a meeting was held between the primary Investigator and the two statistical analysts (JCJ and CO), where differences in the methods used in the analysis was resolved and agreement on relevant changes to the dataset was made (see section on “Retrospective changes to the dataset”).

Final and published analysis were made on the 10^th^ of March 2020 by CO (approved by JCJ).

***Comments to statistical analysis report***

The original plan to do logistic regression or regression and further imputation of missing data was done according to our statistical analysis plan by CO. JCJ was uncertain that the analysis would be valid due to the missing data and low numbers (patients with no adverse events), risk of splitting in the data and, therefore, used a Fischer’s exact test for testing differences between groups. This was accepted at the meeting on the 28^th^ of February 2020. The imputation of data was likewise discussed by the group, since CO had followed the statistical analysis plan and made imputations as worst case and best case and JCJ found that these imputations were not fair to the data. First edition of the analysis by both statisticians can be found below.

| Section/Topic | Item No | Checklist item | Reported on page No |
| --- | --- | --- | --- |
| Title and abstract | | | |
|  | 1a | Identification as a pilot or feasibility randomised trial in the title | 1 |
|  | 1b | Structured summary of pilot trial design, methods, results, and conclusions (for specific guidance see CONSORT abstract extension for pilot trials) | 2 |
| Introduction | | | |
| Background and objectives | 2a | Scientific background and explanation of rationale for future definitive trial, and reasons for randomised pilot trial | 3 |
|  | 2b | Specific objectives or research questions for pilot trial | 3 |
| Methods | | | |
| Trial design | 3a | Description of pilot trial design (such as parallel, factorial) including allocation ratio | 4 |
|  | 3b | Important changes to methods after pilot trial commencement (such as eligibility criteria), with reasons | NA |
| Participants | 4a | Eligibility criteria for participants | 4 |
|  | 4b | Settings and locations where the data were collected | 4 |
|  | 4c | How participants were identified and consented | 4 |
| Interventions | 5 | The interventions for each group with sufficient details to allow replication, including how and when they were actually administered | 5 |
| Outcomes | 6a | Completely defined prespecified assessments or measurements to address each pilot trial objective specified in 2b, including how and when they were assessed | 5-6 |
|  | 6b | Any changes to pilot trial assessments or measurements after the pilot trial commenced, with reasons | NA |
|  | 6c | If applicable, prespecified criteria used to judge whether, or how, to proceed with future definitive trial | 5 |
| Sample size | 7a | Rationale for numbers in the pilot trial | 6 |
|  | 7b | When applicable, explanation of any interim analyses and stopping guidelines | NA |
| Randomisation: |  |  |  |
| Sequence  generation | 8a | Method used to generate the random allocation sequence | 4-5 |
|  | 8b | Type of randomisation(s); details of any restriction (such as blocking and block size) | 4-5 |
| Allocation  concealment  mechanism | 9 | Mechanism used to implement the random allocation sequence (such as sequentially numbered containers), describing any steps taken to conceal the sequence until interventions were assigned | 4-5 |
| Implementation | 10 | Who generated the random allocation sequence, who enrolled participants, and who assigned participants to interventions | 4-5 |
| Blinding | 11a | If done, who was blinded after assignment to interventions (for example, participants, care providers, those assessing outcomes) and how | 5 |
|  | 11b | If relevant, description of the similarity of interventions | NA |
| Statistical methods | 12 | Methods used to address each pilot trial objective whether qualitative or quantitative | 6 |
| Results | | | |
| Participant flow (a diagram is strongly recommended) | 13a | For each group, the numbers of participants who were approached and/or assessed for eligibility, randomly assigned, received intended treatment, and were assessed for each objective | 6 and Figure 1. |
|  | 13b | For each group, losses and exclusions after randomisation, together with reasons | 6 and Figure 1 |
| Recruitment | 14a | Dates defining the periods of recruitment and follow-up | 4 and 6 |
|  | 14b | Why the pilot trial ended or was stopped | 4 |
| Baseline data | 15 | A table showing baseline demographic and clinical characteristics for each group | Table 1 |
| Numbers analysed | 16 | For each objective, number of participants (denominator) included in each analysis. If relevant, these numbers should be by randomised group | Table 2-4 |
| Outcomes and estimation | 17 | For each objective, results including expressions of uncertainty (such as 95% confidence interval) for any estimates. If relevant, these results should be by randomised group | Table 2-3, supplementary tables, page 6-8 |
| Ancillary analyses | 18 | Results of any other analyses performed that could be used to inform the future definitive trial | Supplementary tables |
| Harms | 19 | All important harms or unintended effects in each group (for specific guidance see CONSORT for harms) | 6-7 and table 3 |
|  | 19a | If relevant, other important unintended consequences | NA |
| Discussion | | | |
| Limitations | 20 | Pilot trial limitations, addressing sources of potential bias and remaining uncertainty about feasibility | 10 |
| Generalisability | 21 | Generalisability (applicability) of pilot trial methods and findings to future definitive trial and other studies | 9 |
| Interpretation | 22 | Interpretation consistent with pilot trial objectives and findings, balancing potential benefits and harms, and considering other relevant evidence | 9-10 |
|  | 22a | Implications for progression from pilot to future definitive trial, including any proposed amendments | 10 |
| Other information | | |  |
| Registration | 23 | Registration number for pilot trial and name of trial registry | 2 |
| Protocol | 24 | Where the pilot trial protocol can be accessed, if available | 4 |
| Funding | 25 | Sources of funding and other support (such as supply of drugs), role of funders | 11 |
|  | 26 | Ethical approval or approval by research review committee, confirmed with reference number | 4 |

**
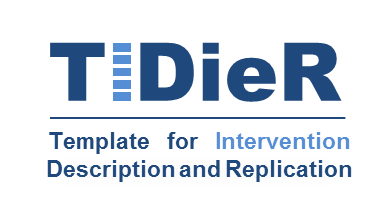
The TIDieR (Template for Intervention Description and Replication) Checklist*:**

Information to include when describing an intervention and the location of the information

| **Item number** | **Item** | **Where located **** | |
| --- | --- | --- | --- |
|  |  | Primary paper  (page or appendix  number) | Other ^†^ (details) |
|  | **BRIEF NAME** |  |  |
| **1.** | Provide the name or a phrase that describes the intervention. | __Page 5_ | ______________ |
|  | **WHY** |  |  |
| **2.** | Describe any rationale, theory, or goal of the elements essential to the intervention. | __Page 3__ | _____________ |
|  | **WHAT** |  |  |
| **3.** | Materials: Describe any physical or informational materials used in the intervention, including those provided to participants or used in intervention delivery or in training of intervention providers. Provide information on where the materials can be accessed (e.g. online appendix, URL). | __Page 4___ | _____________ |
| **4.** | Procedures: Describe each of the procedures, activities, and/or processes used in the intervention, including any enabling or support activities. | __Page 5___ | _____________ |
|  | **WHO PROVIDED** |  |  |
| **5.** | For each category of intervention provider (e.g. psychologist, nursing assistant), describe their expertise, background and any specific training given. | __Page 5___ | _____________ |
|  | **HOW** |  |  |
| **6.** | Describe the modes of delivery (e.g. face-to-face or by some other mechanism, such as internet or telephone) of the intervention and whether it was provided individually or in a group. | __Page 5__ | _____________ |
|  | **WHERE** |  |  |
| **7.** | Describe the type(s) of location(s) where the intervention occurred, including any necessary infrastructure or relevant features. | __Page 5__ | _____________ |
|  | **WHEN and HOW MUCH** |  |  |
| **8.** | Describe the number of times the intervention was delivered and over what period of time including the number of sessions, their schedule, and their duration, intensity or dose. | __Page 5___ | _____________ |
|  | **TAILORING** |  |  |
| **9.** | If the intervention was planned to be personalised, titrated or adapted, then describe what, why, when, and how. | __NA____ | _____________ |
|  | **MODIFICATIONS** |  |  |
| **10.^ǂ^** | If the intervention was modified during the course of the study, describe the changes (what, why, when, and how). | __NA___ | _____________ |
|  | **HOW WELL** |  |  |
| **11.** | Planned: If intervention adherence or fidelity was assessed, describe how and by whom, and if any strategies were used to maintain or improve fidelity, describe them. | ___Page 5-6___ | _____________ |
| **12.^ǂ^** | Actual: If intervention adherence or fidelity was assessed, describe the extent to which the intervention was delivered as planned. | __Page 6-7___ | _____________ |

** **Authors** - use N/A if an item is not applicable for the intervention being described. **Reviewers** – use ‘?’ if information about the element is not reported/not sufficiently reported.

† If the information is not provided in the primary paper, give details of where this information is available. This may include locations such as a published protocol or other published papers (provide citation details) or a website (provide the URL).

ǂ If completing the TIDieR checklist for a protocol, these items are not relevant to the protocol and cannot be described until the study is complete.

* We strongly recommend using this checklist in conjunction with the TIDieR guide (see *BMJ* 2014;348:g1687) which contains an explanation and elaboration for each item.

* The focus of TIDieR is on reporting details of the intervention elements (and where relevant, comparison elements) of a study. Other elements and methodological features of studies are covered by other reporting statements and checklists and have not been duplicated as part of the TIDieR checklist. When a **randomised trial** is being reported, the TIDieR checklist should be used in conjunction with the CONSORT statement (see [www.consort-statement.org](http://www.consort-statement.org)) as an extension of **Item 5 of the CONSORT 2010 Statement.** When a **clinical trial** **protocol** is being reported, the TIDieR checklist should be used in conjunction with the SPIRIT statement as an extension of **Item 11 of the SPIRIT 2013 Statement** (see [www.spirit-statement.org](http://www.spirit-statement.org)). For alternate study designs, TIDieR can be used in conjunction with the appropriate checklist for that study design (see [www.equator-network.org](http://www.equator-network.org)).
