# Supplementary figures and images for: Early Orthostatic Exercise by Head-Up Tilt With Stepping vs. Standard Care After Severe Traumatic Brain Injury Is Feasible
Source: Front Neurol. 2021 Apr 14;12:626014. doi: 10.3389/fneur.2021.626014 (PMC8079637; doi:10.3389/fneur.2021.626014)

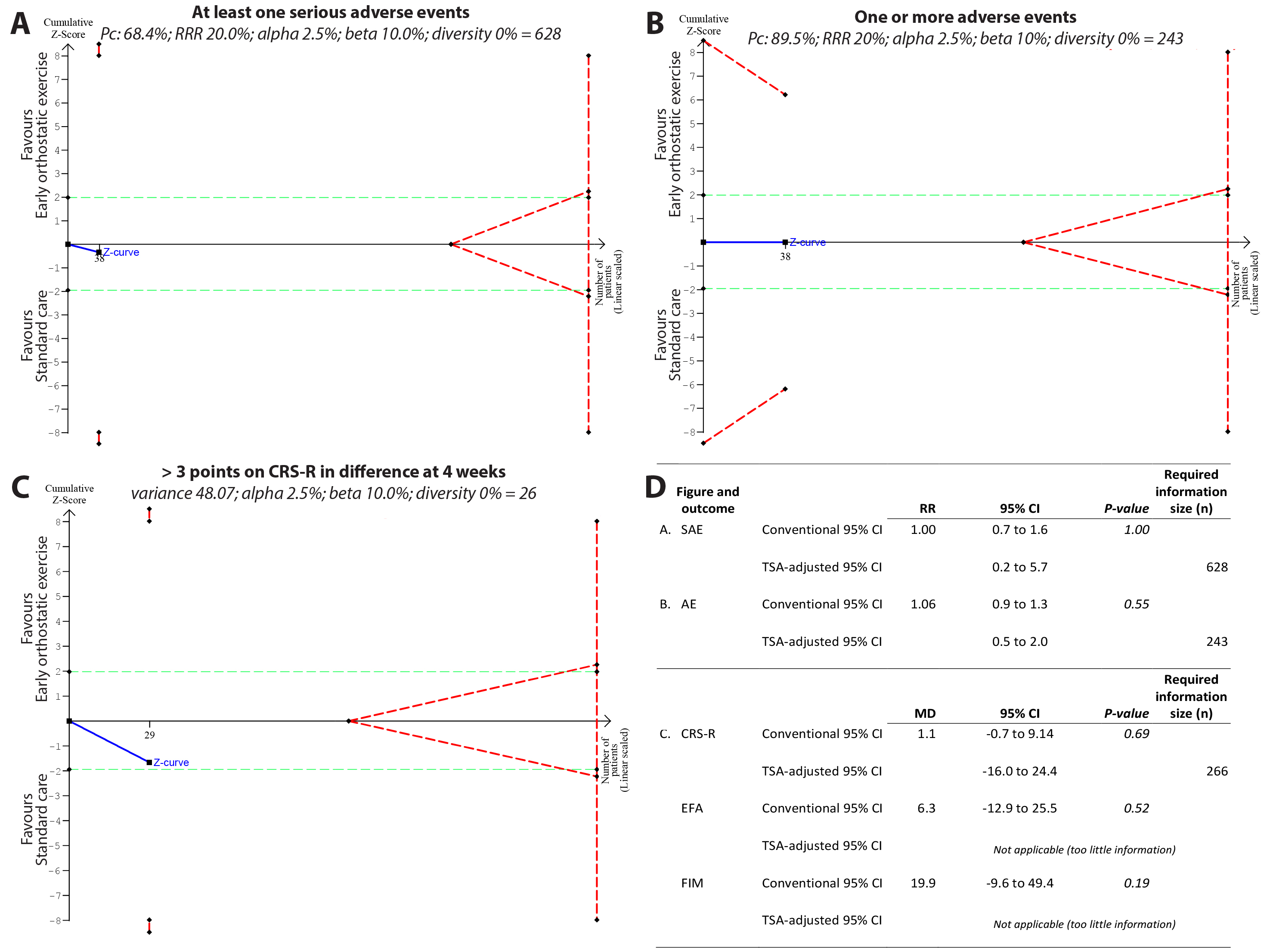

Supplement: Supplementary file 2 [file Image_1.TIFF]
